# Social Pharmacy and Clinical Pharmacy—Joining Forces

**DOI:** 10.3390/pharmacy4010001

**Published:** 2015-12-22

**Authors:** Anna Birna Almarsdottir, Anne Gerd Granas

**Affiliations:** 1 Department of Public Health, Clinical Pharmacology, J.B. Winsløws Vej 19, DK-5000 Odense C, Denmark; 2 Faculty of Health Sciences, Department of Life Sciences and Health, Oslo and Akershus University College of Applied Sciences, P.O. Box 4 St. Olavs plass, N-0130 Oslo, Norway; anne.granas@hioa.no

**Keywords:** clinical pharmacy, social pharmacy, pharmacy education, pharmacy management, pharmacy practice research

## Abstract

This commentary seeks to define the areas of social pharmacy and clinical pharmacy to uncover what they have in common and what still sets them apart. Common threats and challenges of the two areas are reviewed in order to understand the forces in play. Forces that still keep clinical and social pharmacy apart are university structures, research traditions, and the management of pharmacy services. There are key (but shrinking) differences between clinical and social pharmacy which entail the levels of study within pharmaceutical sciences, the location in which the research is carried out, the choice of research designs and methods, and the theoretical foundations. Common strengths and opportunities are important to know in order to join forces. Finding common ground can be developed in two areas: participating together in multi-disciplinary research, and uniting in a dialogue with internal and external key players in putting forth what is needed for the profession of pharmacy. At the end the question is posed, “What’s in a name?” and we argue that it is important to emphasize what unifies the families of clinical pharmacy and social pharmacy for the benefit of both fields, pharmacy in general, and society at large.

## 1. Introduction

“*What’s in a name? That which we call a rose. By any other name would smell as sweet.*”

This reference to William Shakespeare’s play *Romeo and Juliet* is used to imply that the names of things do not affect what they really are. Could this question not just as easily be posed about the disciplines of clinical pharmacy and social pharmacy?

We have experience in working within both clinical and social pharmacy as educators and researchers and, through this, observed the commonalities and differences between these two sister disciplines. The differences have been allowed to dominate the discourse within pharmacy as a profession. We argue that this focus is illogical and detrimental to furthering pharmacy science and practice development.

A simplistic differentiation is that those who entitle themselves clinical pharmacists are practitioners, with little emphasis on research and teaching. Conversely, social pharmacy is an academic university discipline which does not really have a practitioner group with this as a specialization, but has often worked with or supervised community pharmacists. We will argue that this is not a fruitful distinction based on developments within the field of health care.

Our work as researchers and educators has mostly been carried out in the Nordic countries, but we have also sought to incorporate our knowledge of the Anglo-Saxon (North America, UK, and Australia) and European countries outside the Nordic region.

### 1.1. Social Pharmacy Defined

The remit of social pharmacy has been defined by authors from Denmark and the USA [1] as studying…

“*…the drug/medicine sector…from the social scientific and humanistic perspectives. Topics relevant to Social Pharmacy consist of all the social factors that influence medicine use, such as medicine- and health-related beliefs, attitudes, rules, relationships, and processes.*”

Concepts and nomenclature other than *social pharmacy* are used within Anglo-Saxon literature such as *pharmacy practice* and *pharmaceutical policy*. In North America, pharmacy practice as a research discipline has primarily been carried out by clinical and/or hospital pharmacists. British literature refers to pharmacy practice both as a discipline for primary and secondary care pharmacists. A notable exception is also that researchers in England have written a textbook with the title Social Pharmacy [2] and define the concept as:

“*…social pharmacy is a relatively recent concept with Britain, often subsumed under the generic term “Pharmacy Practice”. Initially it was synonymous with the demography of medicines use and “pharmacoepidemiology” but its remit now extends well beyond drug-use surveillance.*”

The same authors, in a paper from 1993 [3], state that social pharmacy cannot be equated to the sociology of pharmacy but that it explores the…

“*…hitherto neglected social domain in which pharmacy is practised from the practitioner’s social perspective.*”

This differs considerably from the definition put forth by Sørensen *et al.* [1] who see it more broadly from a societal perspective rather than pharmacy as a profession.

The term social pharmacy has been used in Britain and occasionally in North America; however, it has been most widely used in the Nordic context and in other (mostly Eastern) European countries [4]. In the USA, pharmacoepidemiology, pharmacoeconomics, and pharmaceutical policy—all considered part of social pharmacy in Europe—are seen as fields separate from pharmacy practice. The UK tends to include pharmaceutical policy and social pharmacy within the term pharmacy practice.

In addition to the lack of unified terminology, the discipline of social pharmacy has been found lacking consensus and a common understanding of what constitutes the research area [5]. This is quite obvious when studying the definitions put forth and the lack of literature about what constitutes the field of social pharmacy.

### 1.2. Clinical Pharmacy Defined

Clinical pharmacy has been defined by professional bodies on both sides of the Atlantic. The European Society of Clinical Pharmacy (ESCP) [6] has defined it as:

“*…a health specialty, which describes the activities and services of the clinical pharmacist to develop and promote the rational and appropriate use of medicinal products and devices.*

*Clinical Pharmacy includes all the services performed by pharmacists practicing in hospitals, community pharmacies, nursing homes, home-based care services, clinics and any other setting where medicines are prescribed and used.*”

The American College of Clinical Pharmacy (AACP) [7] put forth both a long and an abridged definition. The latter states that clinical pharmacy is:

“*…defined as that area of pharmacy concerned with the science and practice of rational medication use.*”

Additionally, the AACP in their unabridged definition emphasize that clinical pharmacy must be engaged in research to contribute to the generation of new knowledge that advances human health and quality of life and that clinical pharmacists should include research as part of their work [7].

Sørensen *et al.* [1], who discussed the definition of social pharmacy, said that

“*Clinical Pharmacy serves as a bridge that overlaps with and connects the natural sciences and Social Pharmacy.*”

### 1.3. Aims

In this article, we will explicitly dig into what defines these two areas of clinical and social pharmacy, what they have in common and what still sets them apart. We aim to challenge researchers, educators, and practitioners relating to either of these fields to find a common ground and thereby strengthen their impact in society.

## 2. Results and Discussion

### 2.1. Threats and Challenges from the Environment

#### 2.1.1. Other Healthcare Professions

Some healthcare providers feel that they have a strong knowledge about medicines, yet this level of knowledge does not match the level of pharmacists. For example, in Norway, nurses specializing in geriatric nursing take over many tasks that clinical pharmacists currently perform or could perform due to their expert knowledge of pharmaceuticals. In Denmark, some of the regions have decided to put the responsibility on general practitioners to do medicine reviews for their patients where pharmacists play only a minor administrative support role. The rise of the related medical specialty of clinical pharmacology [8] has both been a help and a hindrance as the medical counterparts have either been very willing to cooperate or have worked openly to oppose clinical pharmacy from advancing in the healthcare system [9].

#### 2.1.2. A Wide Range of Knowledge

Increased focus on medicines management for nurses and medical doctors can be seen as a natural progression since they increasingly deal with a heavy drug burden, particularly in the elderly [10]. Such a natural progression to fulfill the needs of patients does not follow for pharmacists. The pharmacy profession as a whole is not sufficiently patient-focused to break new ground, being more occupied with the intricacies of systems of drug distribution (*i.e.*, product-focused) [11,12].

Another important challenge lies in the fact that clinical and social pharmacy span a wider range than other disciplines within pharmacy, such as pharmaceutics and medicinal chemistry. This leads them to be more fragmented and have a harder time covering what is needed to teach within these areas [13]. Researchers addressing the topics within social pharmacy have major challenges due to the many different areas of investigation and application [1].

#### 2.1.3. Weak Profession

During the last decades, legal and structural pharmacy ownership reforms in three of the Nordic countries have shown that the pharmacy profession is somewhat weak and divided [14]. Consequently, attempts to increase professionalism within community pharmacies were easily foiled by new commercial owners. Clinical pharmacy was mostly absent from these legal and structural reforms and discussions. One can only wonder how the clinical pharmacy discipline could have contributed to the discourse by arguing for a more distinct role of the pharmacist in safe and effective medicine use in the primary community pharmacy sector.

#### 2.1.4. Favorable Job Market

One important common threat to both clinical and social pharmacy in many countries is a very favorable job market for pharmacists. In Norway, for instance, community pharmacy chains are in great need of labor. Practically every pharmacist can get employment without making efforts either to update their knowledge or to work with patients on their medicine use, *i.e.*, practicing pharmaceutical care and taking responsibility for the patient’s drug-related needs [15].

The challenge an abundant and fragmented job market poses is two-fold. On the one hand, there is no push to develop the field of clinical pharmacy in the primary care sector. On the other hand, pharmacists lose even more ground to de-professionalization in community pharmacies due to increasing proportions of the revenue being linked to beauty and well-being products. In countries with a strong pharmacy industry employing many pharmacists, the competitive environment can shift quickly, leaving pharmacists with the challenge of job loss.

#### 2.1.5. Understanding the Environment

It is therefore imperative to understand the forces in play. To counteract powers shifting the professional services over to commercial well-being products, clinical and social pharmacy should collaborate on increasing understanding and preparing to meet new challenges. Some of the most important new challenges are: the aging of the population and increased complexity in medicine use and polypharmacy; shortened hospital stays coupled with an aging population, which make a smoother sector transition more imperative than ever before; increased cultural diversity challenging the health information and education of patients; increasing use and focus on high-cost drugs (biologicals) in society and the rational use of these by prescribers and patients. These new challenges increase the need for more clinical pharmacy skills in primary care and the increased collaboration of pharmacists across sectors with each other and with other health care professionals.

### 2.2. Forces within Pharmacy Keeping Clinical and Social Pharmacy Apart

#### 2.2.1. Pharmacy Schools

University structures are traditionally divided into faculties, institutes and departments. Internal politics and a lack of understanding for the common ground sometimes limit or even counteract the collaboration of clinical and social pharmacy, thus weakening both fields. The disciplines should be aware of how the healthcare system perceives pharmacists’ abilities to contribute to patient care. Political and organizational aspects, pharmacist-patient communication, and inter-professional communication can be studied using social pharmacy approaches within the “territory” of clinical pharmacy. If this understanding is strong, the clinical pharmacy projects have a greater chance of being accepted. Thus, research in social pharmacy can help to study the important opportunities and challenges facing clinical pharmacy within healthcare. If this understanding is lacking, it makes research of clinical pharmacy services within healthcare difficult.

#### 2.2.2. The Push to Do Research

Social pharmacy is first and foremost a research discipline without a practitioner group with this as a specialization. Social pharmacists, however, have often worked with or supervised community pharmacists. Clinical pharmacy is primarily constituted by practitioners with less emphasis on research and teaching. For the medical profession, however, it has become vital, as clinicians in the hospital environment must publish research results in peer-reviewed journals to be recognized as a strong specialty inside the profession. This trend is also visible within primary care and general practice medicine. It is therefore a weakness that most clinical pharmacists do not do research as a regular part of their practice or only carry out in-house development projects. Even though projects may result in posters presented at practitioner conferences, they do not get published for the wider health professional research arena. Many pharmacists take post-graduate degrees in clinical pharmacy. These degrees focus primarily on becoming better practitioners with less emphasis on research. Combining a PhD and clinical work is increasingly becoming more common, but still not to the extent seen in the profession of medicine.

#### 2.2.3. Priorities in Hospital Pharmacy

Hospital pharmacy in most countries still prioritizes logistic distribution of drugs above clinical pharmacy services. This is quite natural, as the income of most hospital pharmacies depends solely on this area and they are not necessarily remunerated specifically for providing clinical services. Notably, American clinical pharmacists have often been paid specifically for services by the “users” themselves (*i.e.*, the hospital wards), which has made for growth in the USA [16]. Another important and related leap was taken by US hospital pharmacists at the Hilton Head Consensus Conference in 1985 where hospital pharmacy made the unified decision that hospital pharmacies should function as clinical departments with a mission of fostering the appropriate use of medicines. This was a very important idea because most hospital pharmacists thought in terms of adding discrete clinical services rather than conceptualizing the totality of the department’s work as a clinical enterprise [17].

Cross-financing of clinical services with revenues from drug sales and drug distribution naturally slows down the expansion of clinical services to hospital wards [18]. As the hospital pharmacy managers have this focus, there is no impetus to prioritize research—let alone encourage clinical pharmacists on staff to enter PhD programs or to seek adjunct faculty positions at universities. In contrast, medical doctors neither have to excuse themselves for performing research nor have to provide evidence that they are cost-effective in their professional role.

### 2.3. Key Differences between Clinical and Social Pharmacy

Historically there are clear differences with respect to at least four facets of these two disciplines: the levels of study within pharmaceutical sciences, the location where the research has been conducted, the choice of designs and methods, and the use of theories.

- Levels of study within pharmaceutical sciences: The research questions in clinical pharmacy are often at the pharmacological organ-level or study particular pharmacokinetic and pharmacodynamic questions. In social pharmacy, the focus has been more on groups and society at large.
- Location: As clinical pharmacy had its infancy in hospitals, its research was also primarily conducted within this realm. Traditionally, Nordic research within these two fields has been divided so that social pharmacy researchers study community pharmacy with a stronger academic profile, whereas clinical pharmacists study hospital pharmacy and are less inclined to connect directly with faculties of pharmacy.
- Choice of research designs and methods: There have been some differences in preferred research designs between clinical and social pharmacy research. Clinical pharmacy primarily focused on the efficacy and safety of selected medicines or pharmacology-related research questions. Researchers have therefore been prone to using randomized trials and quantitative methodology in general. Social pharmacy research, on the other hand, has used a broader palette of designs and methods from the social sciences.
- Theoretical and methodological foundations: Lastly, clinical pharmacy research has, to a very small extent, been based on theoretical foundations other than biological or epidemiological models. Social pharmacy, in contrast, has historically leaned more towards a social science theoretical approach and a range of qualitative research methods.

### 2.4. Common Strengths and Opportunities

As academics and researchers, we currently observe that the traditional differences between clinical pharmacy and social pharmacy are shrinking. Gradually, clinical pharmacists recognize the need to expand their repertoire of research designs, methods, and theories in order to understand the organizations and the patients they work with. Social pharmacy needs to broaden the research activities to studying pharmacy as it is practiced in a wider context—including institutional pharmacy. Increasingly, hospitals and communities are no longer two separate worlds. It is currently vital to study the patients as they move across the healthcare system, be it in hospitals, home care or primary care.

With the emphasis on medicine use by patients, a clinical mindset does not preclude pharmacists from understanding medicine use at higher levels than the individual patient. There is a need for an evidence base for the use which comes through knowledge about randomized controlled trials (RCT) and pharmacoepidemiology. To increase effective and safe patient care, pharmacoepidemiology and pharmacovigilance can define the problems clinical pharmacy should tackle. In order to operate in times of cost-cutting, clinical pharmacists also need to have a strong understanding of the organization and society they operate within. It is therefore unnatural to make the distinction between social pharmacy and clinical pharmacy when studying how pharmacy makes inroads into new clinical territories.

A prime example of the importance of understanding the environment comes from the hospital pharmacy enterprise in the Central Norway Regional Health Authority that has worked strategically to include clinical pharmacists in hospitals [19]. The increased role of pharmacy in patient care can be attributed to those pharmacists who were in charge of forging ahead, armed with a thorough understanding of the system they worked within. They were able to use this knowledge to influence the Regional Health Authority to understand how pharmacists could be used as a drug knowledge resource in hospital services, securing the seamless transfer of medicine information throughout the hospital stay and back into the community.

Pharmacoepidemiology—historically mostly dealt with in social pharmacy research—has recently become relevant also in clinical pharmacy. For instance, many abstracts and a plenary session at the 30th International Society for Pharmacoepidemiology (ISPE) conference addressed clinical pharmacy and patient perspectives in pharmacoepidemiologial research. Similarly, at the latest conference of the International Society for Pharmacoeconomics and Outcomes Research (ISPOR), abstracts regarding clinical pharmacy research had a sizeable presence.

The Nordic countries all have individual level prescription databases generated from prescriptions dispensed in community pharmacies [20]. Others also generate data on multi-dose dispensed drugs to assess prescribing quality [21]. These have been used extensively by social pharmacy researchers to gain insights into the rational use of medicines and clinical pharmacists are starting to utilize the possibilities of performing research using these data [22].

Social pharmacy researchers have performed research on databases and identified problems in the quality and safety of medicine use. Clinical pharmacists are well situated to identify patients who have a need for tailored interventions. Increased collaboration between these two can be instrumental in furthering outcomes research and making it more patient-focused and relevant for healthcare. Clinical pharmacists are seen as patient safety experts when it comes to medications. Their research collaboration with social pharmacy could thus result in the design of “off the rack” solutions for increasing safe medication use in hospitals and in primary care. These types of solutions, when well defined and described, can then be applied to other healthcare professions (technicians or nurses) for routine implementation.

### 2.5. How to Join Forces

We argue that the reconciliation of forces and strengthening of common ground in clinical and social pharmacy can be developed in two main areas:

(1) Research within these two sister areas increasingly requires multi-disciplinary input. The disciplines can combine the strong methodological and theoretical foundation (in social pharmacy) and the strong foothold in practice (evident in clinical pharmacy) to work with medicine, nursing, social sciences, and health services researchers.
(2) The political aspects of the work going on to enhance rational medicine use require both internal and external key actors. Internally they must unite at the institution level (university, hospital, pharmacy, pharmaceutical societies) and put forth what is needed in education, practice, and research. By not having a united front towards external actors deters them from lobbying and speaking in one voice to policy-makers and other professions.

It is imperative that the message about the ability of the profession to take on more responsibility in patient care is not diluted by focusing on what is “different”. We rephrase Harding and Taylor’s [3] view on social pharmacy: that it is not the sociology of pharmacy, but rather pharmacy’s use of the social sciences to further its usefulness to society.

## 3. Conclusions

### What’s in a Name?

It is important to emphasize what unifies the disciplines of social and clinical pharmacy. No longer can it be stated that clinical pharmacy serves as a bridge between social pharmacy and the rest of the pharmaceutical sciences. It increasingly incorporates many of the same research questions, designs, methods, healthcare system interests and professional autonomy issues as social pharmacy. Social pharmacy, on the other hand, has a need to get closer to practice in order to understand and study the important research questions of today.

So, what’s in a name? Juliet argues that it does not matter that Romeo is from her family’s rival house of Montague—that he is named “Montague”. We argue that it is indeed time to unite the families of clinical pharmacy and social pharmacy for the benefit of both fields, pharmacy in general, and society at large.

## References

[1] Sørensen E.W., Mount J.K., Christensen S.T. (2003). The Concept of Social Pharmacy. Chronic Illn..

[2] Nettleton S., Harding G., Taylor K., Harding G., Nettleton S., Taylor K. (1994). The concept and context of social pharmacy. Social Pharmacy—Innovation and Development.

[3] Harding G., Taylor K. (1993). Defining social pharmacy—It needs its own distinct identity. Int. J. Pharm. Pract..

[4] Praznovcova L., Solich J. (1994). Social pharmacy in pharmacy education. Ceska Slov. Farm..

[5] Desselle S.P., Collins C.C., Harrold M.W., Kalis M.M., Quattrocchi E.J. (2002). Consensus within five academic subdisciplines of pharmacy: Progress toward establishing their scientific paradigms. J. Pharm. Teach..

[6] European Society of Clinical Pharmacy What is Clinical Pharmacy?. http://www.escpweb.org/cms/clinical_pharmacy.

[7] American College of Clinical Pharmacy Clinical Pharmacy Defined. http://www.accp.com/about/clinicalPharmacyDefined.aspx.

[8] Aronson J.K. (2010). A manifesto for clinical pharmacology from principles to practice. Br. J. Clin. Pharmacol..

[9] Hansen S.W. (2013). Korrekt medicinering af patienterne er en laegefaglig kerneydelse. Ugeskr. Laeger.

[10] Jyrkkä J., Vartiainen L., Hartikainen S., Sulkava R., Enlund H. (2006). Increasing use of medicines in elderly persons: A five-year follow-up of the Kuopio 75+Study. Eur. J. Clin. Pharmacol..

[11] Strand L.M., Griffenhagen G., Bowles G.C., Penna R.P., Worthen D.B. (2004). Re-Visioning The Profession. Reflections on Pharmacy by the Remington Medalists 1919–2003.

[12] Rosenthal M., Austin Z., Tsuyuki R.T. (2010). Are Pharmacists the Ultimate Barrier to Pharmacy Practice Change?. CPJ.

[13] Alkhateeb F.M., Latif D.A., Adkins R. (2013). The Economic, Social and Administrative Pharmacy (ESAP) Discipline in US Schools and Colleges of Pharmacy. IJMESS.

[14] Traulsen J.M., Almarsdóttir A.B. (1999). No struggle, no strength: How pharmacists lost their monopoly. Soc. Sci. Med..

[15] Hagelia Lange M., Granås A.G. (2003). Apotekbransjen før og etter ny apoteklov. Tidsskr. Nor. Laegeforen..

[16] American Pharmacists Association 2013 Pharmacists: The Solution to Expanding Clinical Services. http://www.pharmacist.com/pharmacists-solution-expanding-clinical-services.

[17] Zellmer W.A., Brown T.R. (2006). Overview of the History of Hospital Pharmacy in the United States. Handbook of Institutional Pharmacy Practice.

[18] Vinson B.E. (1988). Adjustments of distributive and clinical pharmacy services to financial constraints. Am. J. Hosp. Pharm..

[19] Andersen A.H., Wekre L.J., Sund J.K., Sagen Major A.L., Fredriksen G. (2014). Evaluation of implementation of clinical pharmacy services in Central Norway. Eur. J. Hosp. Pharm..

[20] Furu K., Wettermark B., Andersen M., Martikainen J.E., Almarsdottir A.B., Sørensenm H.T. (2010). The Nordic Countries as a cohort for pharmacoepidemiological research. Basic Clin. Pharmacol. Toxicol..

[21] Halvorsen K.H., Granas A.G., Engeland A., Ruths S. (2012). Prescribing quality for older people in Norwegian nursing homes and home care services using multidose dispensed drugs. Pharmacoepidemiol. Drug Saf..

[22] Hedegaard U., Kjeldsen L.J., Pottegård A., Bak S., Hallas J. (2014). Multifaceted intervention including motivational interviewing to support medication adherence after stroke/transient ischemic attack: A randomized trial. Cerebrovasc. Dis. Extra.

